# *In silico* design and experimental validation of a multi-epitope vaccine candidate against *Helicobacter hepaticus* associated chronic liver inflammation

**DOI:** 10.3389/fimmu.2026.1799290

**Published:** 2026-04-30

**Authors:** Liangliang Weng, Zhang Huijie, Ni Man, Liu Yang, Min Cao

**Affiliations:** 1 Department of Infectious Diseases, The Quzhou Affiliated Hospital of Wenzhou Medical University, Quzhou People’s Hospital, Quzhou, Zhejiang, China; 2 The Affiliated Hospital of Xuzhou Medical University, Xuzhou, Jiangsu, China; 3 Department of Nursing, The Quzhou Affiliated Hospital of Wenzhou Medical University, Quzhou People’s Hospital, Quzhou, Zhejiang, China

**Keywords:** cellular immunity, *Helicobacter hepaticus*, immunoinformatics, multi-epitope vaccine candidate, reverse vaccinology, structural vaccinology, toll-like receptor 2

## Abstract

**Background:**

*Helicobacter hepaticus* is an emerging enterohepatic pathogen associated with chronic gastrointestinal and hepatic inflammatory disorders. Despite its clinical relevance, effective vaccination strategies remain unavailable due to antigenic variability, immune evasion, and limited induction of durable cellular immunity.

**Methods:**

An integrated reverse vaccinology and experimental validation approach was employed to design a multi-epitope vaccine against *H. hepaticus*. Two conserved outer membrane proteins, BamA and an organic solvent tolerance protein, were selected based on antigenicity, surface exposure, and safety profiling. Immunodominant cytotoxic and helper T-cell epitopes were predicted, screened for immunogenicity, allergenicity, and toxicity, and assembled into a rationally designed construct incorporating human β-defensin-3 as an adjuvant. Structural modeling, conformational B-cell epitope mapping, molecular docking, molecular dynamics simulations, and in silico immune simulations were performed. The construct was recombinantly expressed in *Escherichia coli* and evaluated through biophysical, antibacterial, and immunological assays.

**Results:**

The vaccine candidate exhibited favorable physicochemical properties, structural stability, and strong binding affinity toward Toll-like receptor 2. In silico immune simulations predicted robust Th1-biased immune responses with effective memory formation. Recombinant expression yielded a soluble, monodisperse protein. Functional assays demonstrated significant inhibition of *H. hepaticus* growth and host cell adhesion. *In vitro* immunogenicity studies revealed strong peripheral blood mononuclear cell proliferation, enhanced CD4^+^ and CD8^+^ T-cell activation, elevated Th1 cytokine secretion, and antigen-specific antibody production.

**Conclusion:**

The proposed multi-epitope vaccine candidate induces coordinated innate and adaptive immune responses and exhibits promising antibacterial activity against *H. hepaticus*. This study provides a comprehensive and translational framework for epitope-based vaccine development and supports further *in vivo* evaluation of the candidate vaccine.

## Introduction

*Helicobacter hepaticus* is an enterohepatic *Helicobacter* species originally identified in the early 1990s as a murine pathogen capable of driving chronic active hepatitis and liver tumorigenesis in susceptible mouse strains. Chronic colonization can persist for months, sustaining inflammatory liver pathology and creating a well-established experimental framework for dissecting infection-driven hepatocarcinogenesis (1). Subsequent work consolidated *H. hepaticus* as a prototypic model organism linking persistent bacterial infection to progressive hepatic inflammation, dysplasia, and neoplasia (2). Although the strength of evidence is highest in animal systems, the discovery of *H. hepaticus* stimulated broader investigation into the potential contribution of *Helicobacter* species to liver disease beyond the stomach (3).

From a translational standpoint, prevention and control of enterohepatic *Helicobacter* infections remain challenging because chronic colonization is supported by immune evasion, niche adaptation, and inflammatory tissue remodeling. In murine models, *H. hepaticus* infection is associated with reproducible hepatitis and increased hepatocellular neoplasms under specific host and environmental conditions, including interactions with co-exposures and background inflammation (4). These features underscore the need for immunologically informed interventions that can reduce bacterial burden and/or blunt pathogenic inflammation at mucosal and hepatic sites without relying exclusively on antimicrobial therapy.

A rational vaccine strategy for *H. hepaticus* should prioritize antigens that are accessible to host immunity, conserved and essential for bacterial survival, and capable of inducing coordinated cellular and humoral responses. Reverse vaccinology provides a genome-to-antigen discovery framework that addresses these requirements by screening pathogen proteomes to identify vaccine candidates without requiring antigen purification as the first step (5). Over the past two decades, this paradigm has expanded into “reverse vaccinology 2.0,” integrating high-throughput immunology and structural insights to better define protective epitopes and optimize immunogen design (6). In parallel, immunoinformatics-driven multi-epitope vaccine engineering has matured into a practical approach for assembling defined cytotoxic T-lymphocyte (CTL), helper T-lymphocyte (HTL), and B-cell targets into a single construct to broaden population coverage while reducing off-target allergenicity and toxicity risks (7).

Outer membrane biogenesis pathways in Gram-negative bacteria are particularly attractive for vaccine targeting because they govern surface architecture and viability. The β-barrel assembly machinery (BAM) complex is central to the folding and insertion of β-barrel outer membrane proteins, making its key components functionally constrained and broadly conserved (8). BamA, the core BAM subunit, has therefore emerged as an appealing immunological target; antibodies against BamA can exhibit functional activity, supporting its relevance as a vaccine antigen class (9). In addition to such essential surface-associated factors, bacterial proteins that contribute to membrane stability and stress tolerance may provide complementary vaccine targets by impairing colonization fitness and persistence when neutralized or opsonized.

Beyond antigen selection, the adjuvant and innate-sensing context strongly shape vaccine immunogenicity and polarization. Toll-like receptor 2 (TLR2) is a central pattern-recognition receptor involved in sensing bacterial ligands and orchestrating downstream inflammatory and adaptive responses, making it a relevant innate target for antibacterial vaccine design (10). The TLR2 ectodomain is capable of direct interaction with bacterial lipopeptide-like ligands, providing a mechanistic basis for leveraging TLR2-linked immunostimulation in engineered vaccines (11). Host defense peptides, including human β-defensin-3, are of particular interest because they combine immunomodulatory functions with the ability to enhance antigen presentation and immune activation, motivating their use as built-in adjuvant modules in recombinant vaccine constructs (12).

Despite growing adoption of in silico vaccine design pipelines, a persistent limitation in the field is the “validation gap,” where computationally optimized constructs are not consistently advanced through experimentally anchored expression, biophysical profiling, and functional immunogenicity testing. Recent immunoinformatics literature emphasizes that the credibility of multi-epitope vaccines is strengthened when population coverage, structural plausibility, and immune simulation are complemented by laboratory verification of antigen quality and biological activity (13). Accordingly, simulation platforms such as C-ImmSim are often used to forecast immune kinetics and memory formation, but these predictions are most meaningful when interpreted alongside experimental readouts (14).

From a clinical and translational perspective, the development of effective preventive strategies against *H. hepaticus* is of considerable importance. Persistent colonization by enterohepatic Helicobacter species has been implicated in chronic inflammatory conditions and may contribute to progressive liver pathology, particularly in susceptible hosts (3, 4). Current therapeutic approaches rely primarily on antimicrobial regimens, which are often limited by incomplete eradication, recurrence, and the potential emergence of antibiotic resistance. In this context, vaccine-based interventions offer a promising alternative by providing long-term immunological protection while reducing dependence on antibiotics (5, 6, 11). Moreover, the identification of conserved and surface-exposed antigens, coupled with advances in immunoinformatics and structural vaccinology, enables the rational design of targeted vaccine candidates with improved safety and efficacy profiles (6, 10). Therefore, the present study not only advances fundamental understanding of epitope-based vaccine design but also holds potential translational value for the prevention and control of *H. hepaticus*-associated inflammatory diseases.

In this study, we apply an integrated reverse vaccinology–immunoinformatics framework to design a recombinant multi-epitope vaccine candidate targeting *H. hepaticus*, prioritizing two predicted surface-exposed proteins (including BamA) as antigen sources and assembling screened CTL and HTL epitopes into a single chimeric construct with human β-defensin-3 as an immunostimulatory adjuvant. We further couple structural modeling, receptor docking, molecular dynamics-based interaction assessment, and immune simulation with experimental expression in *E. coli*, purification, biophysical characterization, and *in vitro* functional assays of immunogenicity and antibacterial effects. Collectively, this combined computational–experimental workflow aims to generate a mechanistically motivated and experimentally tractable vaccine prototype against *H. hepaticus*, while providing a reproducible template for vaccine development against other understudied enterohepatic pathogens.

## Materials and methods

### Protein sequence retrieval and antigen screening

Protein sequences of the outer membrane protein assembly factor BamA (UniProt accession Q7VHH5) and the organic solvent tolerance protein (UniProt accession Q7VIK8) from *Helicobacter hepaticus* were retrieved in FASTA format from the UniProt database (15). Antigenicity was predicted using VaxiJen v3.0 under the bacterial model with a default threshold of 0.4, and proteins exceeding this cutoff were considered probable antigens (16). Allergenicity was assessed using AllerTOP v2.1, and proteins predicted to be allergenic were excluded from further analyses. Transmembrane helices and protein topology were predicted using TMHMM v2.0 to identify surface-exposed and extracellular regions, ensuring the selection of immunologically accessible vaccine targets (17).

To ensure rational antigen selection, an initial screening of the *H. hepaticus* proteome was conducted based on key criteria relevant to vaccine design, including antigenicity, subcellular localization, surface accessibility, allergenicity, and functional importance. Proteins predicted to be cytoplasmic, poorly antigenic, or potentially allergenic were excluded from further consideration. Particular emphasis was placed on outer membrane-associated proteins due to their direct exposure to the host immune system and their established relevance as vaccine targets in Gram-negative bacteria.

Among the screened candidates, the outer membrane protein assembly factor BamA and the organic solvent tolerance protein were selected based on their high antigenicity scores, predicted surface exposure, non-allergenic nature, and functional roles in membrane biogenesis and bacterial survival. BamA, as a central component of the β-barrel assembly machinery, is highly conserved and essential, reducing the likelihood of antigenic variation and immune escape. Similarly, the organic solvent tolerance protein contributes to membrane stability under stress conditions, supporting bacterial persistence.

### Prediction and screening of T-cell epitopes

Cytotoxic T lymphocyte (CTL) and helper T lymphocyte (HTL) epitopes were predicted using tools available through the Immune Epitope Database (IEDB) platform (18). CTL epitopes were identified using NetMHCpan-EL 4.1, and peptides with a percentile rank ≤1 were classified as strong MHC class I binders. HTL epitopes were predicted using NetMHCIIpan-EL 4.1, and peptides with a percentile rank <10 were selected as high-affinity MHC class II binders (19).

All predicted epitopes were further evaluated for antigenicity using VaxiJen v3.0, allergenicity using AllerTOP v2.1, and toxicity using ToxinPred. Only epitopes predicted to be immunogenic, non-allergenic, and non-toxic were retained for inclusion in the vaccine candidate (20).

### Population coverage analysis

Global and regional population coverage of the selected CTL and HTL epitopes was assessed using the IEDB Population Coverage Tool. This analysis integrates HLA allele frequency distributions across diverse ethnic and geographical populations to estimate the proportion of individuals likely to mount an immune response against the selected epitope set, thereby evaluating the potential global applicability of the designed vaccine construct (21).

### Multi-epitope vaccine construct design

Selected CTL and HTL epitopes were assembled into a multi-epitope vaccine construct designed to elicit robust cellular and humoral immune responses. Human β-defensin-3 was incorporated at the N-terminus as an immunostimulatory adjuvant and linked to the first epitope using a rigid EAAAK linker to ensure appropriate structural separation. CTL epitopes were joined using AAY linkers to enhance proteasomal processing and MHC class I presentation, whereas HTL epitopes were connected using GPGPG linkers to preserve epitope flexibility and facilitate efficient MHC class II recognition (22). The final construct was reassessed for antigenicity and allergenicity to confirm its suitability for downstream analyses.

### Physicochemical characterization of the vaccine construct

Physicochemical properties of the vaccine construct, including molecular weight, theoretical isoelectric point (pI), instability index, aliphatic index, and grand average of hydropathicity (GRAVY), were computed using the ProtParam tool available on the ExPASy server (23). Protein solubility and aggregation propensity were predicted using SoluProt and CamSol to assess expression feasibility and structural stability in heterologous expression systems.

### Backbone dynamics and intrinsic disorder prediction

Residue-level backbone dynamics and intrinsic disorder were predicted using the DynaMine server. Residues with scores >0.80 were classified as rigid, values between 0.69 and 0.80 as context-dependent flexible, and scores <0.69 as intrinsically disordered. This analysis provided insight into structural flexibility and potential antigenic regions relevant for immune recognition (24).

### Tertiary structure prediction and validation

The three-dimensional structure of the vaccine construct was predicted using AlphaFold3. Structural validation was performed using PROCHECK to generate Ramachandran plots and evaluate stereochemical quality. The distribution of residues in favored and allowed regions was used to assess the reliability and structural integrity of the predicted model (25).

### Secondary structure analysis

Secondary structural elements, including α-helices, β-strands, and random coils, were predicted using the PDBsum server to provide insight into folding patterns and structural regions likely to influence epitope exposure and immunogenicity (26).

### Prediction of conformational B-cell epitopes

Discontinuous (conformational) B-cell epitopes were predicted using the ElliPro server based on the refined three-dimensional structure of the vaccine construct. Predicted epitope clusters were ranked according to protrusion index (PI), with higher PI values indicating increased surface accessibility and immunogenic potential (27).

### Molecular docking and interaction analysis

Protein–protein docking between the vaccine construct and Toll-like receptor 2 (TLR2) was performed using the ClusPro server to evaluate innate immune receptor engagement. The top-ranked docked complex was analyzed using PDBsum to identify hydrogen bonds, salt bridges, and hydrophobic interactions at the binding interface (28).

### Molecular dynamics simulations and binding free energy calculations

Molecular dynamics (MD) simulations were conducted using the AMBER software package to investigate the stability and interaction dynamics of the vaccine–TLR2 complex. The AMBER ff19SB force field was applied to the receptor, and GAFF2 parameters were used for the vaccine construct. The system was solvated in a TIP3P water box with a 12 Å buffer and neutralized with Na^+^ and Cl^-^ ions. Energy minimization was followed by equilibration at 298 K and 1 bar using a 2 fs timestep. Production simulations were performed, and trajectory frames were recorded at 10 ps intervals. Structural stability was evaluated using RMSD, RMSF, and radius of gyration analyses, while principal component analysis (PCA) was used to characterize dominant conformational motions. Binding free energies were calculated using MM-GBSA and MM-PBSA approaches (29).

### *In silico* immune simulation

Immune response simulations were performed using the C-ImmSim server to evaluate the immunogenic potential of the multi-epitope vaccine candidate. The simulation was conducted using default immune system parameters, with three antigen injections administered at time steps 1, 84, and 168, corresponding to a typical prime–boost vaccination schedule with four-week intervals. Each time step represents 8 hours of real biological time.

The total simulation duration was set to 350 time steps to capture both primary and secondary immune responses, as well as the development of immunological memory. The simulation volume was maintained at 10, and the random seed was fixed to ensure reproducibility.

Additional parameters included standard settings for lymphocyte population dynamics, antigen processing, and cytokine responses. Output data were generated for antibody titers, cytokine profiles, B-cell and T-cell populations, and memory cell formation (30). These results were analyzed to assess the ability of the vaccine candidate to induce sustained and robust immune responses.

### Codon optimization and *in silico* cloning

The amino acid sequence of the vaccine construct was reverse-translated using EMBOSS Backtranseq. Codon optimization for *E. coli* (strain K-12) was performed using the ExpOptimizer tool to enhance expression efficiency. Restriction sites for NdeI and XhoI were introduced at the 5′ and 3′ ends, respectively, and the optimized gene was cloned in silico into the pET-28a(+) expression vector using SnapGene software to confirm correct insertion and orientation (31).

### Experimental validation

#### Gene synthesis and cloning

The final 187-amino-acid multi-epitope vaccine (MEV) sequence, incorporating the β-defensin-3 adjuvant and EAAAK, AAY, and GPGPG linkers, was codon-optimized for expression in *E. coli* K-12. The synthetic gene was commercially synthesized and cloned into the pET-28a(+) expression vector under the control of a T7 promoter with an N-terminal six-histidine tag. Recombinant plasmids were transformed into *E. coli* BL21(DE3).

#### Recombinant protein expression and purification

A single transformed colony was cultured in LB medium supplemented with kanamycin (50 μg/mL) at 37 °C until the optical density at 600 nm reached 0.6–0.8. Protein expression was induced with 0.5 mM IPTG, and cultures were incubated at 18 °C for 16–18 h to promote soluble expression. Cells were harvested by centrifugation, lysed by sonication, and clarified lysates were subjected to Ni²^+^-NTA affinity chromatography. Eluted fractions were analyzed by SDS-PAGE, pooled, and dialyzed against phosphate-buffered saline (PBS, pH 7.4).

#### Size exclusion chromatography and dynamic light scattering

Purified vaccine protein was analyzed by size exclusion chromatography using a Superdex 200 Increase 10/300 GL column connected to an ÄKTA FPLC system to assess purity, oligomeric state, and aggregation behavior. Monodisperse fractions were further analyzed by dynamic light scattering (DLS) to confirm homogeneity and structural stability.

#### Preparation of vaccine-induced antibodies

To obtain vaccine-induced antibodies, peripheral blood mononuclear cells (PBMCs) isolated from healthy donors were stimulated *in vitro* with the purified multi-epitope vaccine construct (10 μg/mL) for 5–7 days under standard culture conditions. Cell culture supernatants containing secreted antibodies were collected and clarified by centrifugation at 10,000 × g for 10 min to remove cellular debris. The resulting supernatants, enriched in antigen-specific antibodies, were either used directly for functional assays or concentrated using centrifugal filtration units with a 10 kDa molecular weight cutoff.

For assays requiring higher purity, immunoglobulin fractions were optionally purified using Protein G affinity chromatography according to standard protocols. The concentration of antibodies was determined using a NanoDrop spectrophotometer, and aliquots were stored at −80 °C until use.

### *In vitro* antibacterial and immunogenicity assays

#### Bacterial culture and antibacterial activity

*Helicobacter hepaticus* (ATCC 51449) was cultured under microaerophilic conditions on Columbia agar supplemented with 5% sheep blood. Growth inhibition assays were performed by incubating bacterial suspensions with sera obtained from vaccine-stimulated immune cells, followed by colony-forming unit enumeration.

#### Adhesion inhibition assay

AGS human gastric epithelial cells were infected with *H. hepaticus* pre-incubated with PBMC-derived, vaccine-induced antibodies obtained as described above. Adherent bacteria were quantified by CFU assay after removal of non-adherent cells.

#### *In vitro* immunogenicity

Peripheral blood mononuclear cells (PBMCs) were isolated from heparinized blood of healthy adult donors by Ficoll-Paque density gradient centrifugation. Cells were washed twice with phosphate-buffered saline (PBS) and resuspended in RPMI-1640 medium supplemented with 10% fetal bovine serum, 1% penicillin-streptomycin, and 2 mM L-glutamine. PBMCs were seeded in 24-well or 96-well plates at an appropriate density and stimulated with purified multi-epitope vaccine protein (10 μg/mL). Unstimulated cells served as the negative control, while cells treated with phytohemagglutinin (PHA) or anti-CD3/CD28 were used as the positive control where applicable. Cultures were maintained at 37 °C in a humidified 5% CO2 incubator.

#### Lymphocyte proliferation assay

PBMC proliferation was assessed after 72 h of stimulation using a CCK-8 or MTT-based colorimetric assay according to the manufacturer’s instructions. Absorbance was measured using a microplate reader, and the stimulation index was calculated relative to the unstimulated control.

#### Cytokine measurement

Culture supernatants were collected after 48–72 h of stimulation and analyzed for IFN-γ, IL-2, and TNF-α levels using commercially available ELISA kits according to the manufacturers’ protocols.

#### T-cell activation analysis

For flow cytometric analysis, stimulated PBMCs were harvested, washed, and stained with fluorophore-conjugated antibodies against CD3, CD4, CD8, and activation markers such as CD69 or CD25. Samples were acquired on a flow cytometer and analyzed to determine the proportions of activated CD4^+^ and CD8^+^ T cells.

#### Antigen-specific antibody detection

To evaluate antibody responses generated *in vitro*, culture supernatants collected from vaccine-stimulated PBMCs were analyzed by indirect ELISA. Briefly, 96-well plates were coated with purified vaccine protein, blocked with bovine serum albumin, incubated with collected supernatants, and then probed with HRP-conjugated anti-human IgG. Optical density was measured at 450 nm.

### Statistical analysis

All experiments were performed in triplicate. Data are presented as mean ± standard deviation. Statistical analyses were conducted using one-way analysis of variance (ANOVA) followed by appropriate *post hoc* tests, and p values <0.05 were considered statistically significant.

## Results

### Identification of conserved surface-exposed antigenic proteins

**Figure 1:**
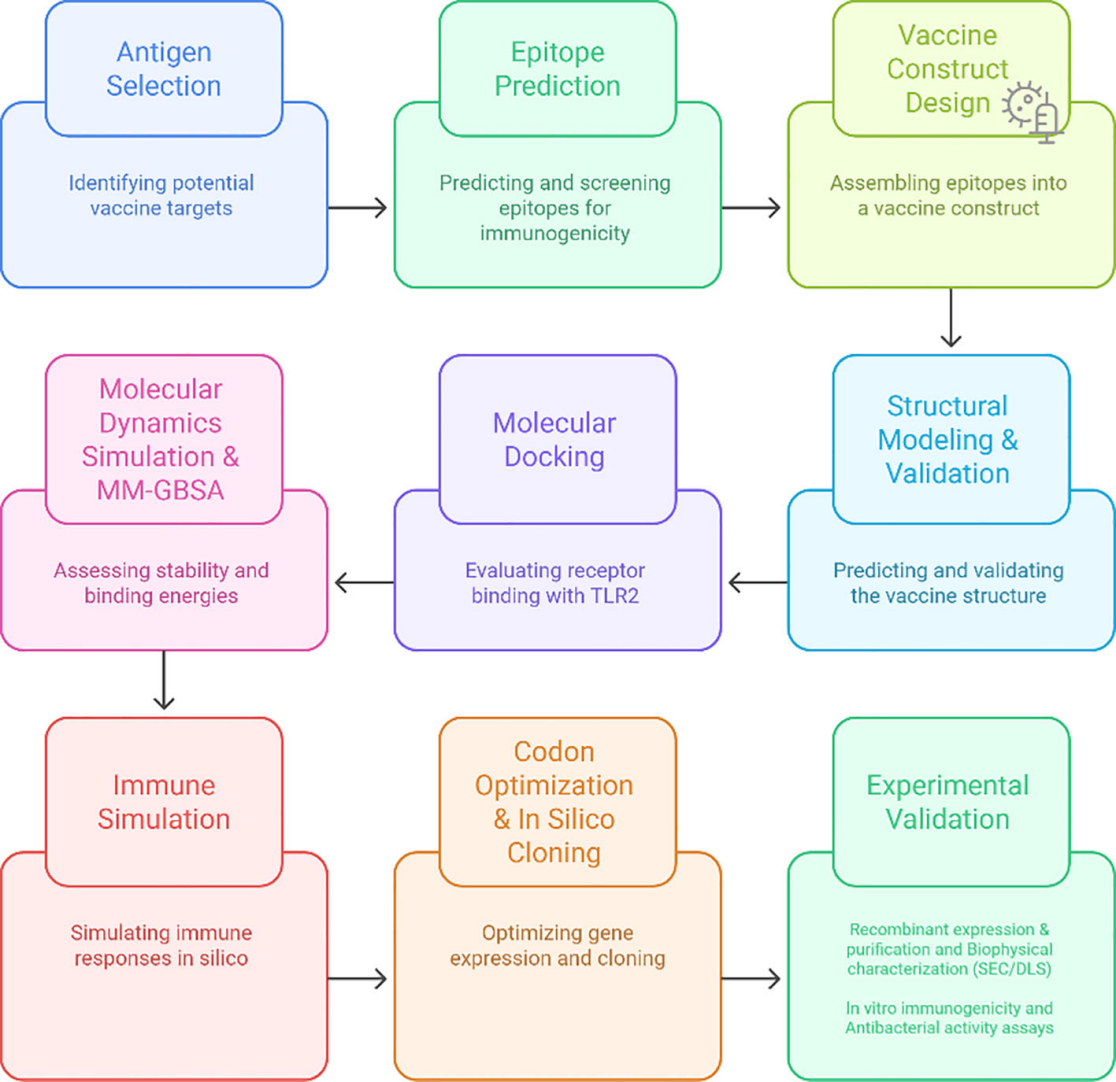
Integrated workflow for the design and experimental validation of the multi-epitope vaccine candidate against *H. hepaticus*. The workflow includes antigen identification and screening, epitope prediction and filtering, vaccine construct design, structural and computational analyses (including modeling, docking, molecular dynamics, and immune simulation), followed by recombinant expression, biophysical characterization, and *in vitro* functional validation.

A reverse vaccinology–based screening strategy was employed to identify surface-exposed and immunologically relevant proteins from *H. hepaticus*. Two outer membrane–associated proteins, the outer membrane protein assembly factor BamA (UniProt: Q7VHH5) and the organic solvent tolerance protein (UniProt: Q7VIK8), emerged as optimal vaccine targets. Both proteins exhibited strong antigenicity scores using the VaxiJen v3.0 bacterial model (0.66 and 1.00, respectively) and were predicted to be non-allergenic by AllerTOP v2.1 (**Table 1**; **Figure 1**).

**Table 1:** Surface-exposed antigenic proteins selected for multi-epitope vaccine design.

Protein name	UniProt accession	Function	Antigenicity score (VaxiJen)	Allergenicity	TM helix region	Predicted localization
Outer membrane protein assembly factor BamA	Q7VHH5	β-barrel protein assembly and outer membrane biogenesis	0.66	Non-allergen	1–745	Outer membrane
Organic solvent tolerance protein	Q7VIK8	Enhances membrane stability and solvent resistance	1.00	Non-allergen	1–710	Outer membrane

Antigenicity was predicted using VaxiJen v3.0 (bacterial model). Allergenicity was assessed using AllerTOP v2.1. Transmembrane topology and localization were predicted using TMHMM v2.0.

Transmembrane topology analysis using TMHMM v2.0 revealed that both proteins possess extensive extracellular regions with limited transmembrane helices, supporting their accessibility to host immune recognition **Supplementary Figure 2**). Importantly, BamA plays a critical role in β-barrel protein assembly and outer membrane biogenesis, while Q7VIK8 contributes to membrane stability under stress conditions. The essential and conserved nature of these proteins underscores their suitability as vaccine antigens and reduces the likelihood of immune escape through antigenic variation.

### Selection of immunogenic and safe T-cell epitopes with broad HLA coverage

From the selected antigenic proteins, T-cell epitope prediction identified five cytotoxic T-lymphocyte (CTL) epitopes and four helper T-lymphocyte (HTL) epitopes with high immunogenic potential. Epitope selection was stringently filtered to retain only peptides predicted to be immunogenic, non-allergenic, and non-toxic **Supplementary Table 4**). Several epitopes demonstrated maximal immunogenicity scores (1.00), including the CTL epitope LVIRTILEY and the HTL epitopes AYAITFNVNQGENII and LFSGALYKYTSNAID.

**Figure 2:**
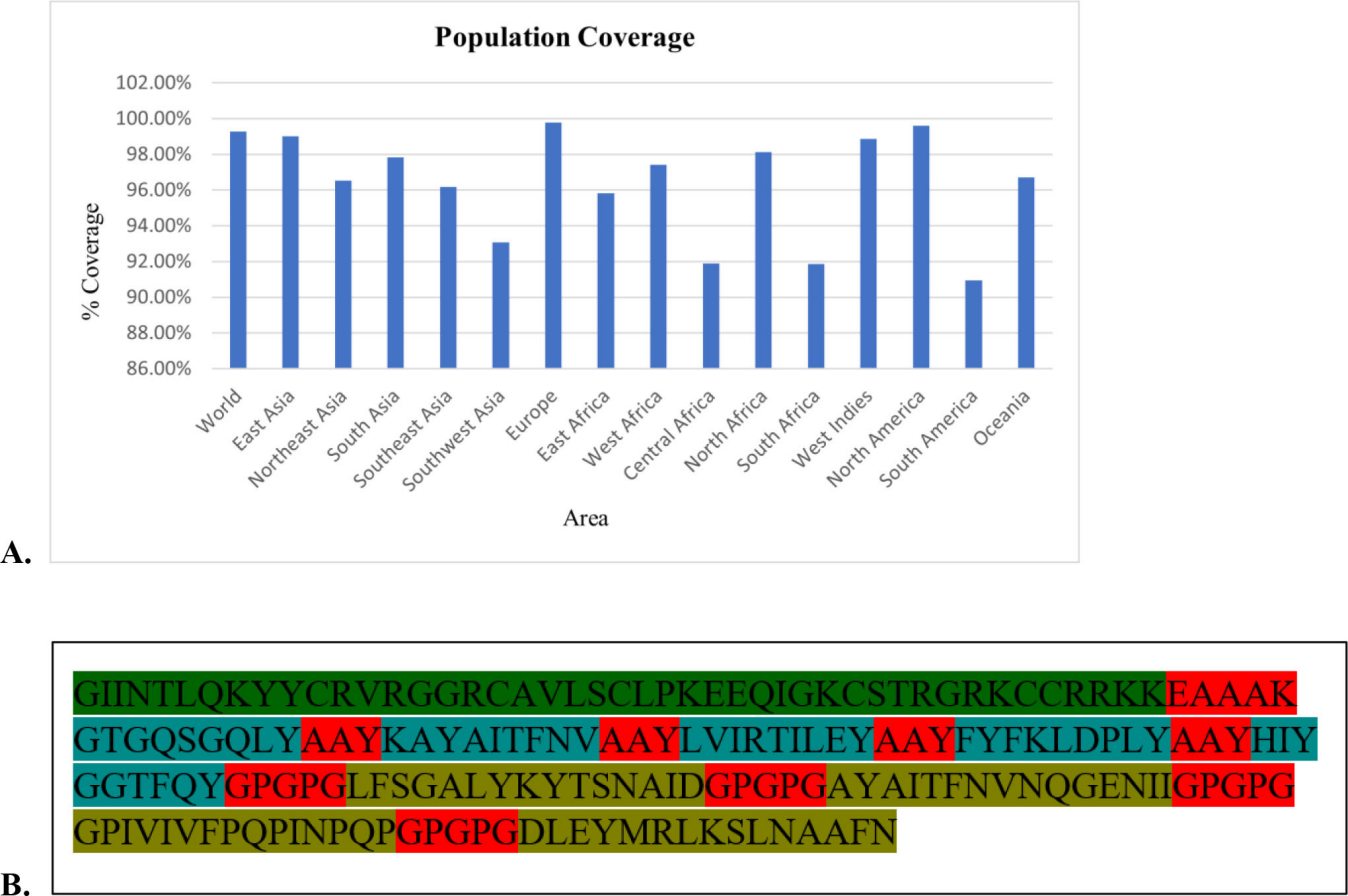
Population coverage analysis and schematic representation of the multi-epitope vaccine construct. **(A)** Global population coverage of selected CTL and HTL epitopes calculated using the IEDB population coverage tool, indicating broad HLA allele representation. **(B)** Linear schematic of the vaccine construct showing the arrangement of CTL and HTL epitopes linked by AAY and GPGPG linkers, respectively, with human β-defensin-3 incorporated as an adjuvant via an EAAAK linker.

The selected epitopes exhibited strong binding affinity to multiple prevalent HLA class I and class II alleles, suggesting the capacity to elicit robust cellular immune responses across genetically diverse populations. Comprehensive HLA allele distributions are provided in **Supplementary Table 1**. Population coverage analysis further demonstrated extensive global applicability, with a cumulative coverage of 99.29% (**Figure 2A**), highlighting the translational potential of the vaccine construct.

### Design and physicochemical characterization of the multi-epitope vaccine construct

The finalized multi-epitope vaccine construct comprised 187 amino acids and incorporated five CTL epitopes and four HTL epitopes, joined using immunologically optimized AAY and GPGPG linkers to ensure efficient antigen processing and presentation. Human β-defensin-3 was fused at the N-terminus as an immunostimulatory adjuvant via an EAAAK linker to maintain structural independence (**Figure 2B**).

**Figure 3:**
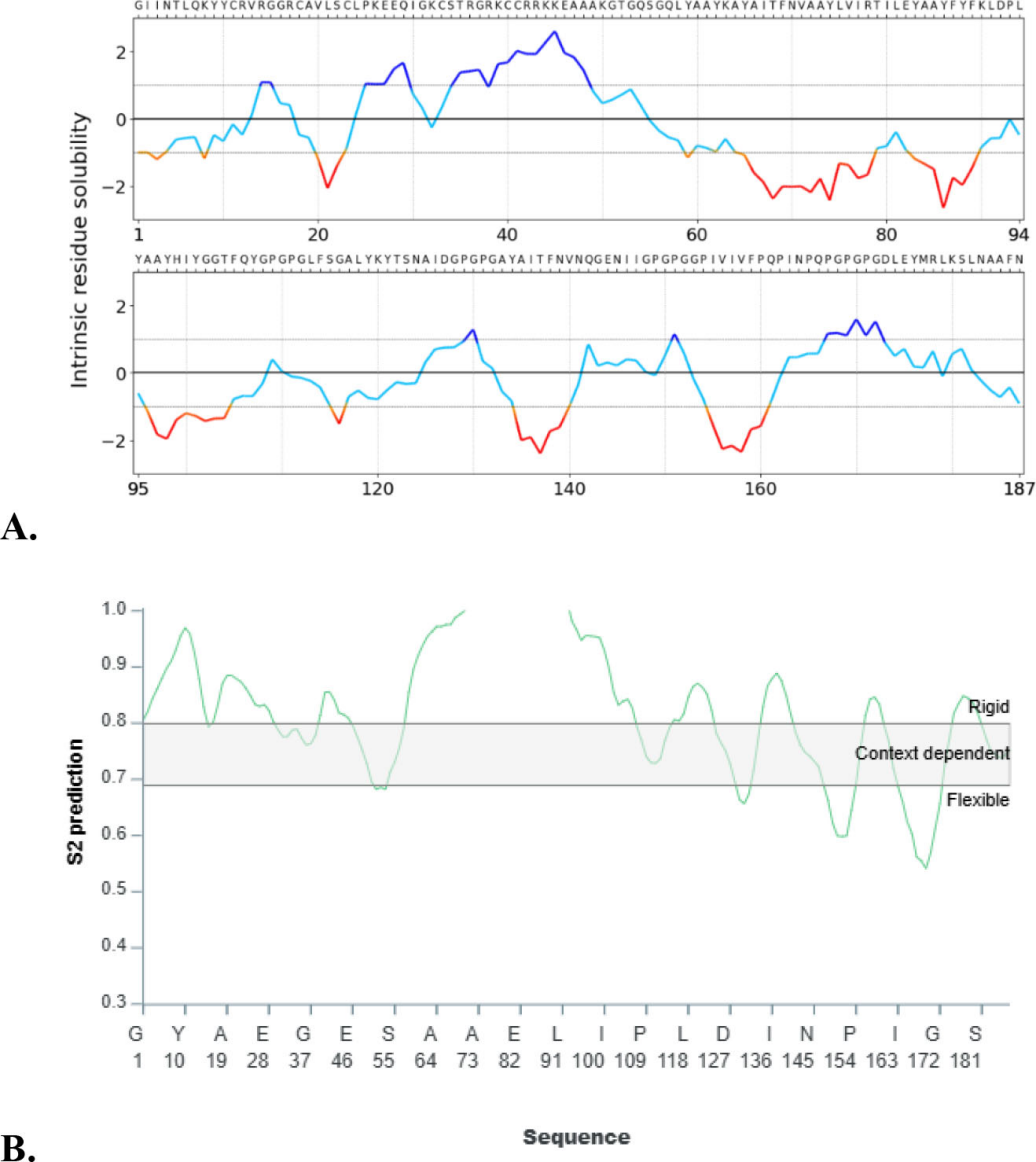
Population coverage and structural organization of the multi-epitope vaccine candidate. **(A)** Global population coverage of selected CTL and HTL epitopes calculated using the IEDB population coverage tool. **(B)** Schematic representation of the multi-epitope vaccine construct showing the arrangement of cytotoxic T-lymphocyte (CTL) and helper T-lymphocyte (HTL) epitopes. Human β-defensin-3 is incorporated at the N-terminus as an adjuvant and is linked via an EAAAK linker. CTL epitopes are connected using AAY linkers, while HTL epitopes are joined using GPGPG linkers. All functional components, including epitopes, linkers, and adjuvant regions, are clearly annotated.

Physicochemical analysis revealed a molecular weight of 20.23 kDa and a theoretical isoelectric point (pI) of 9.41, indicative of favorable biochemical properties for recombinant expression (**Table 2**). The instability index (39.52) classified the construct as stable, while a negative GRAVY value (−0.144) suggested an overall hydrophilic nature. A high solubility score (0.808) further supported the feasibility of heterologous expression and downstream experimental handling (**Figure 3**).

**Table 2:** Physicochemical properties and key design features of the multi-epitope vaccine construct.

Parameter	Value
Construct length	187 amino acids
Molecular weight	20.23 kDa
Theoretical pI	9.41
Instability index	39.52 (stable)
Aliphatic index	77.86
GRAVY	−0.144
Solubility score	0.808
CTL epitopes	5
HTL epitopes	4
Adjuvant	Human β-defensin-3
Linkers	EAAAK, AAY, GPGPG
Global population coverage	99.29%
Best docking score (TLR2)	−1104 kcal/mol
MD simulation duration	500 ns
Expression host	*E. coli* K-12

Physicochemical properties were calculated using ProtParam and SoluProt. Population coverage was estimated using the IEDB population coverage tool.

### Structural modeling, validation, and conformational B-cell epitope prediction

Three-dimensional structural modeling using AlphaFold3, followed by refinement with GalaxyRefine, yielded a well-ordered and compact vaccine structure. Ramachandran plot analysis confirmed favorable stereochemical quality, with the majority of residues located within favored and allowed regions (**Supplementary Figure 3B**). Secondary structure analysis revealed a balanced distribution of α-helices, β-strands, and random coils, supporting structural integrity and epitope accessibility (**Supplementary Figure 3C**).

**Figure 4:**
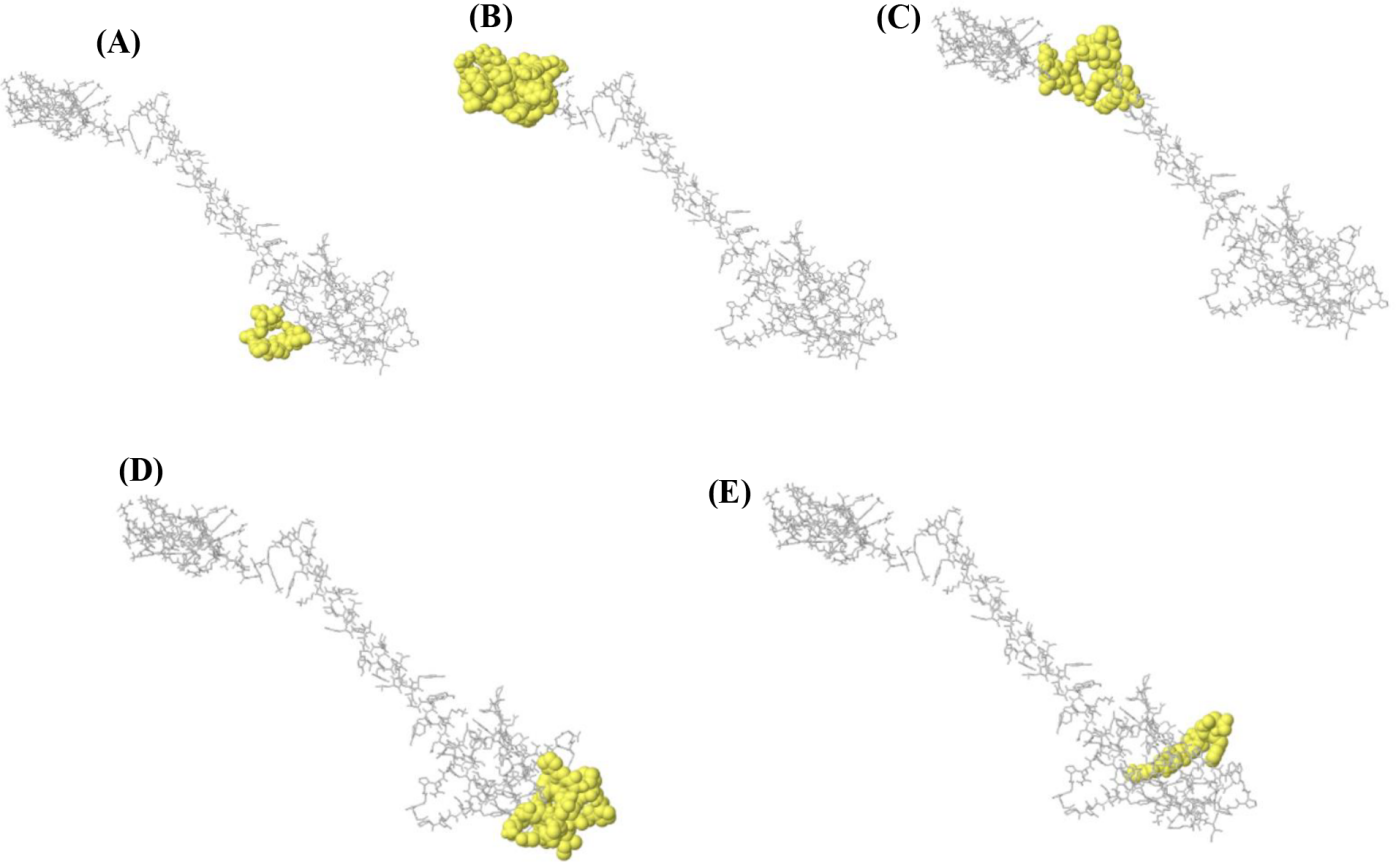
Molecular docking poses of the multi-epitope vaccine construct with Toll-like receptor 2 (TLR2). Representative docked conformations **(A–E)** illustrating different binding orientations of the vaccine construct (yellow surface representation) on the TLR2 receptor (gray backbone representation).

Based on the refined structure, five conformational (discontinuous) B-cell epitopes were predicted using ElliPro (**Table 3**>). These epitopes exhibited high protrusion index values (up to 0.793), indicative of strong surface exposure. Three-dimensional mapping confirmed that the predicted epitopes were predominantly located on solvent-accessible regions of the construct (**Figure 4**), supporting their potential to elicit humoral immune responses.

**Table 3:** Predicted conformational (discontinuous) B-cell epitopes of the vaccine construct.

Epitope ID	Residue range	No. of residues	Protrusion index (PI)
Epitope 1	166–175	10	0.793
Epitope 2	1–40	38	0.785
Epitope 3	46–64	18	0.731
Epitope 4	107–154	23	0.577
Epitope 5	180–187	8	0.555

Conformational B-cell epitopes were predicted using ElliPro based on the refined three-dimensional structure. Complete residue compositions are provided in <xr rid="st2">**Supplementary Table 2**</xr>.

### Molecular docking and dynamics demonstrate stable engagement with TLR2

**Figure 5:**
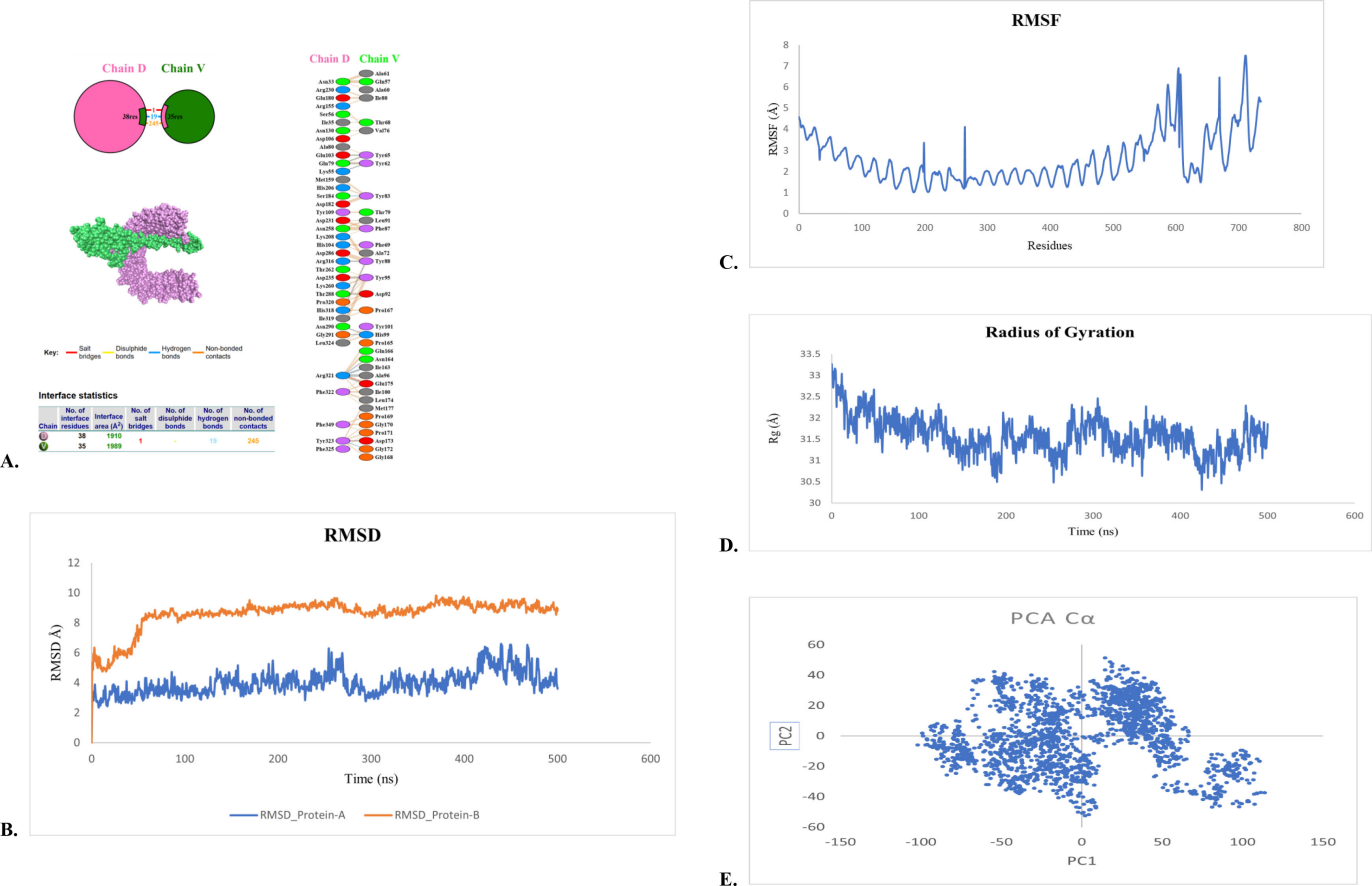
Molecular docking and interaction analysis of the vaccine construct with Toll-like receptor 2 (TLR2). Docking was performed using the ClusPro server. **(A)** Predicted docked complex showing binding orientation. **(B)** Close-up view of the interaction interface highlighting hydrogen bonds and hydrophobic interactions. **(C–E)** Molecular dynamics simulation analyses showing root mean square deviation (RMSD), radius of gyration, and residue fluctuation (RMSF), indicating structural stability of the complex over time.

Protein–protein docking analysis revealed a strong interaction between the vaccine construct and Toll-like receptor 2 (TLR2), a key pattern recognition receptor involved in antibacterial immune signaling. The top-ranked docked complex displayed extensive interfacial contacts, including hydrogen bonds and hydrophobic interactions, consistent with stable receptor engagement (**Figure 5A**).

To further evaluate interaction stability, 500 ns molecular dynamics simulations were performed. RMSD and radius of gyration analyses demonstrated minimal structural deviation and sustained compactness throughout the simulation period (**Figures 5B–D**). Residue-level fluctuation analysis indicated limited flexibility at the binding interface, while principal component analysis revealed constrained collective motions. MM-GBSA binding free energy calculations showed a progressive stabilization of the complex, with ΔG_bind improving from −159.82 kcal/mol at 0 ns to −172.46 kcal/mol at 500 ns (**Table 4**), confirming a thermodynamically favorable interaction.

**Table 4:** MM-GBSA binding free energy components of the vaccine–TLR2 complex.

Simulation time (ns)	ΔG_bind_ (kcal/mol)	Coulomb	Covalent	H-bond	Lipophilic	Solvation (GB)	van der Waals
0	−159.82	−83.47	+40.21	−20.88	−70.65	+85.14	−110.17
500	−172.46	+18.92	−31.54	−41.76	−47.02	+24.63	−95.69

Binding free energies were calculated using MM-GBSA following 500 ns molecular dynamics simulations of the vaccine–TLR2 complex.

### In silico immune simulation predicts robust and durable immune responses

**Figure 6:**
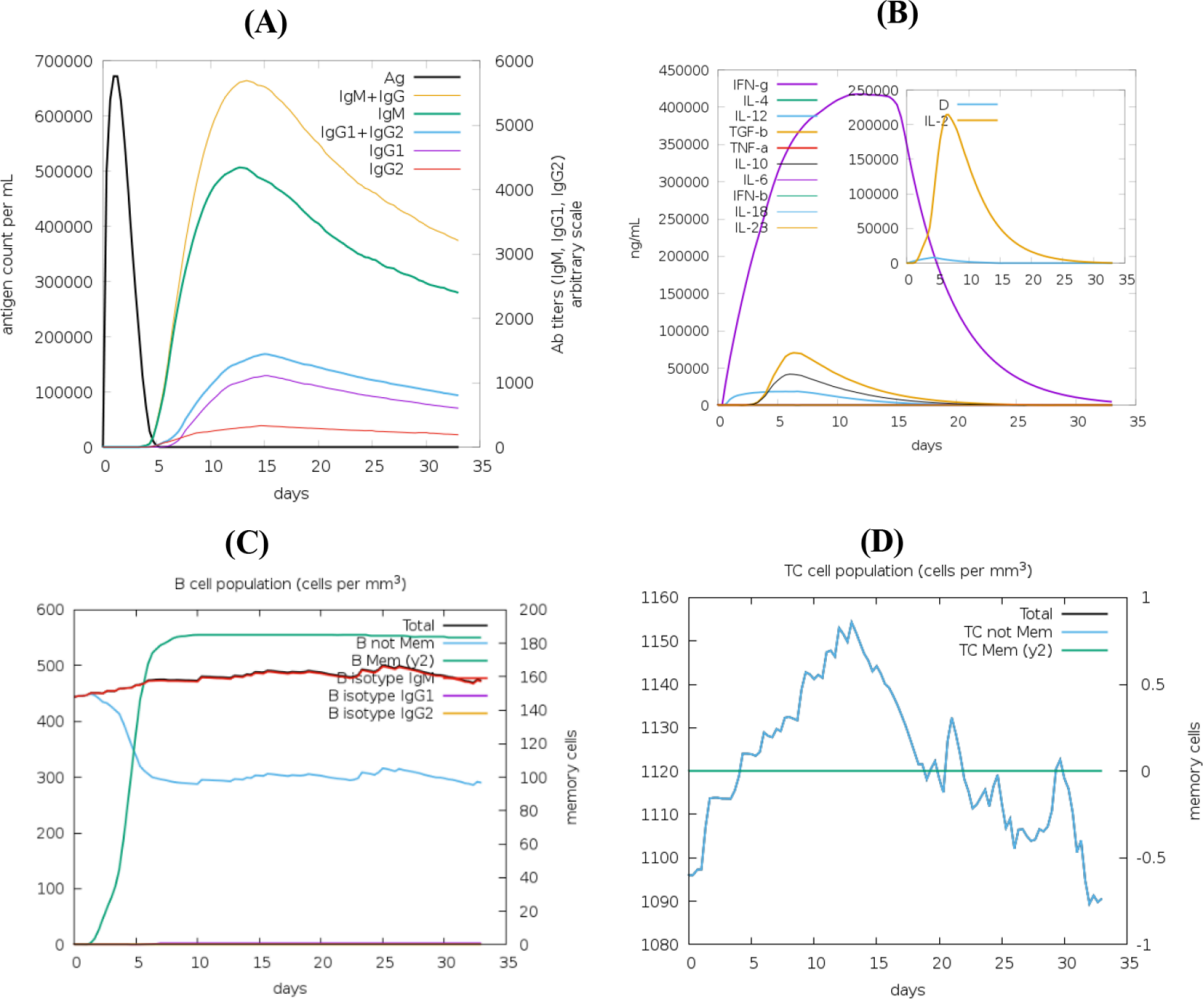
In silico immune simulation of the vaccine construct using the C-ImmSim server. **(A)** Antigen clearance and antibody response over time. **(B)** Cytokine profile showing IFN-γ and IL-2 production. **(C)** Memory B-cell population dynamics. **(D)** CD8^+^ T-cell response indicating activation and expansion following simulated vaccination.

Agent-based immune simulations using C-ImmSim predicted effective antigen clearance and sustained antibody production following vaccination (**Figure 6A**). The vaccine construct elicited a pronounced Th1-biased immune response, characterized by elevated IFN-γ and IL-2 levels (**Figure 6B**), along with expansion of memory B-cell populations (**Figure 6C**) and robust CD8^+^ cytotoxic T-cell responses (**Figure 6D**). These findings suggest the capacity of the construct to induce durable adaptive immunity.

### Recombinant expression and biophysical validation confirm construct stability

**Figure 7:**
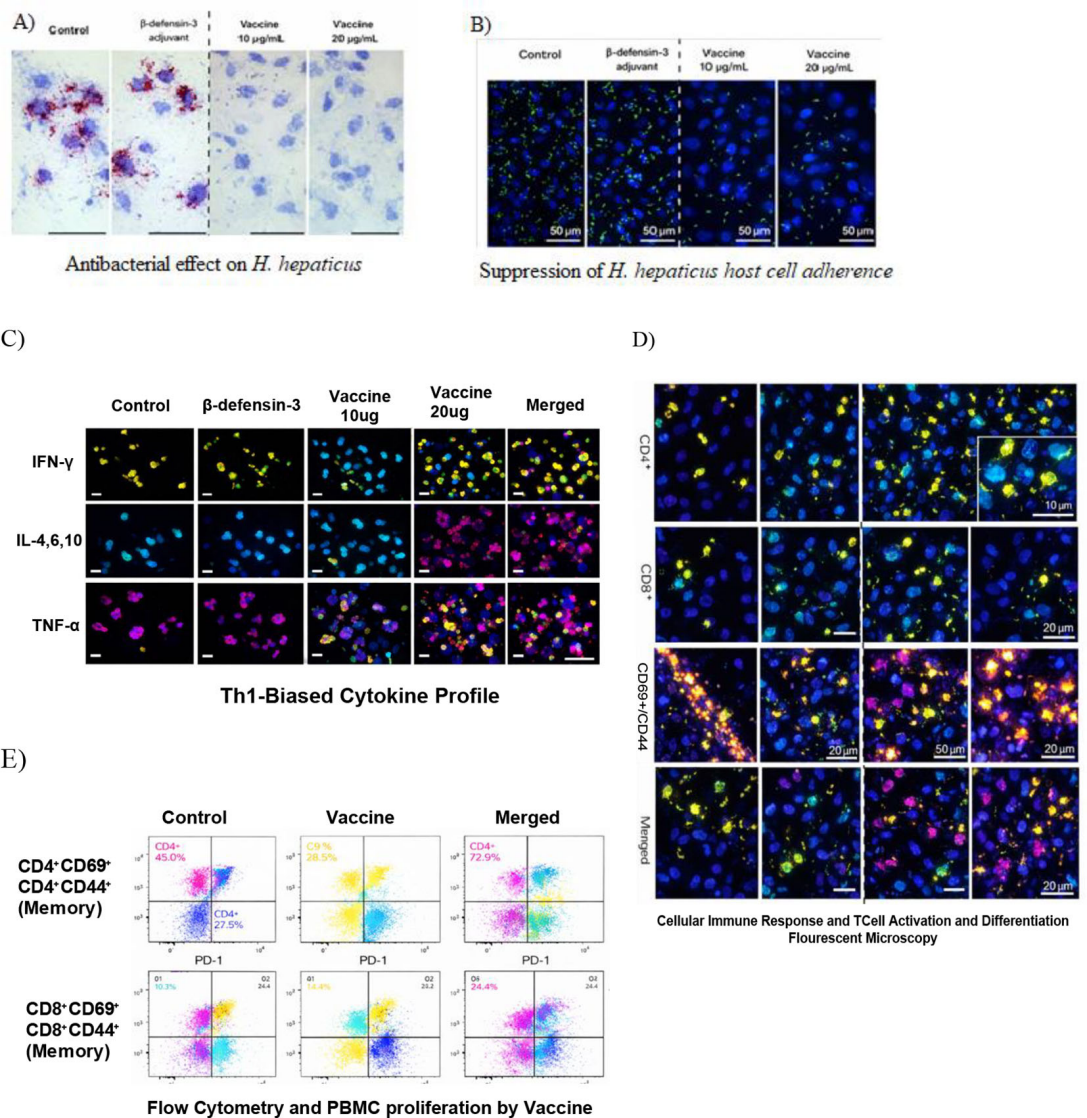
Functional evaluation of the multi-epitope vaccine candidate-induced immune responses against *H. hepaticus*. **(A)** Representative microscopy images showing growth inhibition of *H. hepaticus* following treatment with vaccine candidate-induced antibodies. Quantitative analysis of bacterial inhibition (CFU reduction) is presented as mean ± SD. **(B)** Fluorescence microscopy images showing reduced adhesion of *H. hepaticus* to AGS cells following antibody treatment. Quantitative adhesion data are presented as percentage reduction relative to control. **(C)** PBMC proliferation assay results showing increased lymphocyte proliferation upon stimulation with the vaccine candidate, expressed as stimulation index. **(D)** Flow cytometric analysis showing activation of CD4^+^ and CD8^+^ T cells following stimulation, with quantitative representation of activated cell populations. **(E)** Cytokine production (IFN-γ, IL-2, TNF-α) and antigen-specific IgG levels measured by ELISA. Data are presented as mean ± standard deviation from three independent experiments. Statistical significance was determined using one-way ANOVA (*p < 0.05, **p < 0.01).

The vaccine construct was successfully expressed in *E. coli* BL21 (DE3) using the pET-28a(+) expression system. SDS-PAGE and Western blot analyses confirmed soluble expression and the expected molecular weight of approximately 21 kDa (**Figures 7A, B**). Purification yielded 18.6 ± 1.4 mg/L culture with a final purity exceeding 95% (**Table 5**).

**Table 5:** Recombinant expression, purification, and biophysical characterization of the vaccine protein.

Parameter	Result
Expression host	*E. coli* BL21 (DE3)
Expression vector	pET-28a(+)
Induction conditions	0.5 mM IPTG, 18 °C, 16 h
Solubility	Predominantly soluble
Observed molecular weight	~21 kDa
Purification yield	18.6 ± 1.4 mg/L culture
Final purity	>95%
SEC monomer fraction	91.6 ± 2.3%
DLS hydrodynamic diameter	4.8 ± 0.6 nm
Polydispersity index	0.19 ± 0.04
Aggregation	<5%

Purity was confirmed by SDS-PAGE and densitometry. Size exclusion chromatography and dynamic light scattering analyses confirmed monodispersity and structural stability.

Biophysical characterization further supported structural stability. Size exclusion chromatography revealed a predominant monomeric species (91.6 ± 2.3%), while dynamic light scattering measurements indicated a low polydispersity index (0.19 ± 0.04) and minimal aggregation (<5%) (**Figure 7C**), in agreement with in silico predictions.

### The vaccine induces potent antibacterial activity and immunogenic responses *in vitro*

Functional evaluation demonstrated that sera derived from vaccine-stimulated immune cells significantly inhibited *H. hepaticus* growth (71.0%) and markedly reduced bacterial adhesion to AGS gastric epithelial cells (28.6 ± 3.7%) compared with controls 1111 **Table 6A**). These findings indicate effective antibody-mediated antibacterial activity.

**Table 6:** Functional antibacterial and immunogenic activity of the multi-epitope vaccine.

A. Antibacterial and anti-adhesion activity			
Treatment	Growth inhibition (%)		Adhesion to AGS cells (%)
Untreated control	–		100 ± 4.8
Control serum	6.4		94.2 ± 6.1
β-defensin-3	35.8*		63.5 ± 5.4*
Vaccine-immune serum	71.0*		28.6 ± 3.7*
B. Cellular and humoral immune responses			
Immune parameter	Vaccine response		
PBMC proliferation index (20 µg/mL)	4.5 ± 0.5	
IFN-γ (pg/mL)	612 ± 48	
IL-2 (pg/mL)	487 ± 39	
TNF-α (pg/mL)	401 ± 36	
CD4^+^CD69^+^ T cells (%)	28.6 ± 3.1	
CD8^+^CD69^+^ T cells (%)	24.3 ± 2.7	
Antigen-specific IgG (OD_450_)	2.36 ± 0.18	
IgG1/IgG2 ratio	1.4 ± 0.2	

Data are presented as mean ± SD (n = 3). *p < 0.05, **p < 0.001 compared with control groups.

Immunological assays further revealed strong cellular and humoral immune responses. The vaccine induced robust PBMC proliferation (stimulation index = 4.5 ± 0.5), elevated production of Th1-associated cytokines (IFN-γ, IL-2, and TNF-α), and substantial activation of CD4^+^ and CD8^+^ T-cell populations (**Table 6B**; **Figures 7C–E**). High antigen-specific IgG titers with a balanced IgG1/IgG2 ratio further confirmed effective humoral immunity.

### Overall significance

Collectively, these results demonstrate that the rationally designed multi-epitope vaccine integrates broad HLA coverage, structural stability, favorable innate immune receptor engagement, and potent antibacterial and immunogenic activity. The seamless integration of computational predictions with experimental validation highlights the translational promise of this vaccine platform for combating *H. hepaticus*–associated disease.

## Discussion

The development of effective vaccines against *H. hepaticus* remains challenging due to antigenic variability, immune evasion strategies, and the limited accessibility of conserved surface antigens (3, 4). In this study, a reverse vaccinology-driven strategy was employed to overcome these challenges by integrating antigen screening, epitope-based design, structural vaccinology, and experimental validation. The selection of BamA and the organic solvent tolerance protein as vaccine targets was guided by their essential roles in outer membrane biogenesis and bacterial fitness, surface exposure, and high predicted antigenicity. BamA, in particular, is a conserved and functionally indispensable protein in Gram-negative bacteria and has been increasingly recognized as a promising vaccine target due to its limited sequence variability and essentiality for bacterial survival (32).

The immunological design of the multi-epitope vaccine construct was centered on the inclusion of both cytotoxic and helper T-cell epitopes with broad HLA coverage, a critical factor for ensuring population-wide vaccine efficacy (5, 6). The predicted high global population coverage supports the translational relevance of the construct, particularly given the extensive polymorphism of HLA alleles across human populations (7). The simultaneous incorporation of CTL and HTL epitopes enables coordinated activation of CD8^+^ effector responses and CD4^+^ helper functions, which are essential for sustained cellular immunity and memory formation against persistent bacterial pathogens (33).

Structurally, the rational use of EAAAK, AAY, and GPGPG linkers ensured appropriate spatial separation of functional elements and minimized epitope interference, thereby promoting efficient antigen processing and presentation (34). The incorporation of human β-defensin-3 as an adjuvant further enhanced the immunological profile of the construct by facilitating innate immune activation and bridging innate and adaptive responses (8). Physicochemical analyses, backbone dynamics predictions, and tertiary structure validation collectively demonstrated that the designed construct is stable, soluble, and conformationally well organized. The identification of multiple conformational B-cell epitopes with high surface accessibility further supports the capacity of the vaccine to elicit balanced humoral responses, complementing the dominant T-cell–mediated immunity (35).

Innate immune engagement is a key determinant of vaccine efficacy, and Toll-like receptor 2 (TLR2) plays a central role in recognizing bacterial components and initiating immune signaling cascades (9). The strong interaction observed between the vaccine construct and TLR2, supported by molecular docking and molecular dynamics simulations, suggests effective engagement of innate immune pathways. The stability of the vaccine–TLR2 complex over extended simulation time and favorable binding free energy profiles indicate a persistent and biologically relevant interaction. Such engagement is known to enhance antigen uptake, cytokine production, and downstream adaptive immune priming, thereby amplifying vaccine-induced immune responses (10).

### Experimental validation and functional efficacy

A notable strength of the present study is the experimental validation of computational predictions. Recombinant expression of the vaccine construct in *E. coli* yielded a soluble and predominantly monomeric protein with minimal aggregation, confirming the accuracy of in silico predictions related to stability and solubility. Functional assays demonstrated that sera derived from vaccine-stimulated immune cells significantly inhibited *H. hepaticus* growth and reduced bacterial adhesion to host epithelial cells, indicating effective immune-mediated antibacterial activity. These findings are consistent with previous reports highlighting the importance of antibody-dependent and T-cell–mediated mechanisms in controlling *Helicobacter* infections (10, 11).

*In vitro* immunogenicity analyses further demonstrated robust PBMC proliferation, enhanced activation of CD4^+^ and CD8^+^ T-cell populations, and a Th1-biased cytokine profile characterized by elevated IFN-γ and IL-2 levels. Th1-dominated responses are widely associated with protection against intracellular and persistent bacterial pathogens and are considered desirable for vaccines targeting *Helicobacter* species (36). The observed induction of memory-associated T-cell markers further suggests the potential of the construct to support long-term immune protection.

### Limitations and future perspectives

Despite the promising computational and *in vitro* findings, several limitations should be acknowledged. The protective efficacy, durability of immune memory, and safety profile of the vaccine construct remain to be validated in appropriate *in vivo* models. In addition, formulation strategies, dosing regimens, and delivery platforms may significantly influence immunogenic outcomes and should be explored in future studies. Comparative evaluation against existing vaccine candidates and assessment under physiological infection conditions will be essential steps toward clinical translation (37, 38).

## Conclusion

In conclusion, this study presents a rationally designed, structurally validated, and experimentally supported multi-epitope vaccine candidate against *H. hepaticus*. By integrating reverse vaccinology, structural analysis, immune simulation, and functional validation, the proposed vaccine candidate demonstrates strong potential to elicit robust innate and adaptive immune responses. The comprehensive and translational nature of this approach addresses key limitations of conventional vaccine strategies and provides a valuable framework for the development of epitope-based vaccines against *Helicobacter* species and other challenging bacterial pathogens.

## Data Availability

The original contributions presented in the study are included in the article/**Supplementary Material**. Further inquiries can be directed to the corresponding authors.
