# Supplementary material for: In silico design and experimental validation of a multi-epitope vaccine candidate against Helicobacter hepaticus associated chronic liver inflammation
Source: Front Immunol. 2026 Apr 30;17:1799290. doi: 10.3389/fimmu.2026.1799290 (PMC13171813; doi:10.3389/fimmu.2026.1799290)
Supplement: Supplementary file 1 [file Table1.docx]

# **SUPPLEMENTARY DATA**

## **Supplementary Table S1. Complete HLA allele distribution of selected CTL and HTL epitopes**

### **Supplementary Table S1A. MHC class I (CTL) epitope–HLA allele distribution**

| **Epitope** | **Source protein** | **Immunogenicity** | **Allergenicity** | **Toxicity** | **HLA class I alleles** |
| --- | --- | --- | --- | --- | --- |
| GTGQSGQLY | BamA | 0.66 | Non-allergen | Non-toxic | HLA-A01:01, HLA-A30:02, HLA-B15:01, HLA-A26:01 |
| KAYAITFNV | BamA | 0.66 | Non-allergen | Non-toxic | HLA-A02:06, HLA-A32:01, HLA-A68:02, HLA-A02:01, HLA-A30:01, HLA-B51:01, HLA-A02:03, HLA-B58:01 |
| LVIRTILEY | BamA | 1.00 | Non-allergen | Non-toxic | HLA-B15:01, HLA-A26:01, HLA-A30:02, HLA-B35:01, HLA-A32:01, HLA-B58:01, HLA-A11:01, HLA-A01:01, HLA-B53:01, HLA-B57:01, HLA-A03:01 |
| FYFKLDPLY | Q7VIK8 | 0.66 | Non-allergen | Non-toxic | HLA-A30:02, HLA-A23:01, HLA-A24:02, HLA-B35:01, HLA-B53:01, HLA-A32:01, HLA-A26:01 |
| HIYGGTFQY | Q7VIK8 | 0.66 | Non-allergen | Non-toxic | HLA-A26:01, HLA-A30:02, HLA-B15:01, HLA-B35:01, HLA-A03:01, HLA-A32:01, HLA-A11:01, HLA-A01:01, HLA-A68:01, HLA-B53:01, HLA-A30:01, HLA-B58:01, HLA-B57:01 |

### **Supplementary Table S1B. MHC class II (HTL) epitope–HLA allele distribution**

| **Epitope** | **Source protein** | **Immunogenicity** | **Allergenicity** | **Toxicity** | **HLA-DRB1 alleles** |
| --- | --- | --- | --- | --- | --- |
| AYAITFNVNQGENII | BamA | 1.00 | Non-allergen | Non-toxic | DRB113:02, DRB104:05, DRB107:01, DRB101:01, DRB104:01, DRB103:01 |
| GPIVIVFPQPINPQP | BamA | 0.66 | Non-allergen | Non-toxic | DRB112:01, DRB107:01, DRB101:01, DRB115:01, DRB109:01, DRB113:02, DRB104:01, DRB104:05, DRB1*08:02 |
| DLEYMRLKSLNAAFN | Q7VIK8 | 0.66 | Non-allergen | Non-toxic | DRB101:01, DRB104:01, DRB108:02, DRB111:01, DRB104:05, DRB107:01, DRB1*15:01 |
| LFSGALYKYTSNAID | Q7VIK8 | 1.00 | Non-allergen | Non-toxic | DRB115:01, DRB104:05, DRB107:01, DRB109:01, DRB1*04:01 |

**Footnote:** CTL and HTL epitopes were predicted using NetMHCpan-EL 4.1 and NetMHCIIpan-EL 4.1, respectively. Allergenicity was evaluated using AllerTOP v2.1 and toxicity using ToxinPred.

## **Supplementary Table S2. Complete residue composition of predicted conformational B-cell epitopes**

| **Epitope no.** | **Residues (amino acid positions)** | **No. of residues** | **Protrusion index (PI)** |
| --- | --- | --- | --- |
| 1 | Q166, P167, G168, P169, G170, P171, G172, D173, L174, E175 | 10 | 0.793 |
| 2 | G1, I2, I3, N4, T5, L6, Q7, K8, Y9, Y10, C11, R12, V13, R14, G15, G16, R17, C18, A19, V20, L21, S22, C23, L24, P25, K26, Q29, I30, G31, K32, C33, S34, T35, R36, G37, R38, K39, C40 | 38 | 0.785 |
| 3 | E46, A47, A48, A49, K50, G51, T52, G53, Q54, S55, G56, Q57, L58, Y59, A60, A61, K63, A64 | 18 | 0.731 |
| 4 | Y107, G108, P109, G110, P111, G112, L113, S115, K120, N141, Q142, G143, E144, N145, I146, I147, G148, P149, G150, P151, G152, G153, P154 | 23 | 0.577 |
| 5 | K180, S181, L182, N183, A184, A185, F186, N187 | 8 | 0.555 |

**Footnote:** Discontinuous B-cell epitopes were predicted using ElliPro based on the refined three-dimensional structure of the multi-epitope vaccine construct.

## **Supplementary Table S3. Codon optimization and in silico cloning parameters**

| **Parameter** | **Before optimization** | **After optimization** |
| --- | --- | --- |
| Codon adaptation index (CAI) | 1.00 | 0.75 |
| GC content (%) | 57.58 | 53.30 |
| Expression vector | – | pET-28a(+) |
| Restriction sites | – | NdeI (5′), XhoI (3′) |

**Footnote:** Codon optimization was performed using ExpOptimizer, and cloning was simulated using SnapGene. Optimization parameters indicate suitability for high-level expression in Escherichia coli K-12.

**Supplementary Table S4. Selected cytotoxic (CTL) and helper (HTL) T-cell epitopes with immunogenicity and safety profiles**

| **Epitope** | **Source protein** | **Immunogenicity** | **Allergenicity** | **Toxicity** | **Representative HLA alleles** |
| --- | --- | --- | --- | --- | --- |
| **Cytotoxic T-lymphocyte (CTL) epitopes** | | | | |  |
| GTGQSGQLY | BamA | 0.66 | Non-allergen | Non-toxic | HLA-A01:01; HLA-A30:02; HLA-B15:01 |
| KAYAITFNV | BamA | 0.66 | Non-allergen | Non-toxic | HLA-A02:01; HLA-A68:02; HLA-B58:01 |
| LVIRTILEY | BamA | 1.00 | Non-allergen | Non-toxic | HLA-A03:01; HLA-B35:01; HLA-B57:01 |
| FYFKLDPLY | Q7VIK8 | 0.66 | Non-allergen | Non-toxic | HLA-A24:02; HLA-B35:01 |
| HIYGGTFQY | Q7VIK8 | 0.66 | Non-allergen | Non-toxic | HLA-A30:02; HLA-B58:01 |
| **Helper T-lymphocyte (HTL) epitopes** | | | | |  |
| AYAITFNVNQGENII | BamA | 1.00 | Non-allergen | Non-toxic | DRB1*01:01; DRB1*04:01 |
| GPIVIVFPQPINPQP | BamA | 0.66 | Non-allergen | Non-toxic | DRB1*07:01; DRB1*15:01 |
| DLEYMRLKSLNAAFN | Q7VIK8 | 0.66 | Non-allergen | Non-toxic | DRB1*04:01; DRB1*11:01 |
| LFSGALYKYTSNAID | Q7VIK8 | 1.00 | Non-allergen | Non-toxic | DRB1*15:01; DRB1*04:05 |

**Footnote:** CTL epitopes were predicted using NetMHCpan-EL 4.1 and HTL epitopes using NetMHCIIpan-EL 4.1. Only epitopes predicted as immunogenic, non-allergenic (AllerTOP v2.1), and non-toxic (ToxinPred) were retained. Complete HLA allele distributions are provided in Supplementary Table S1.


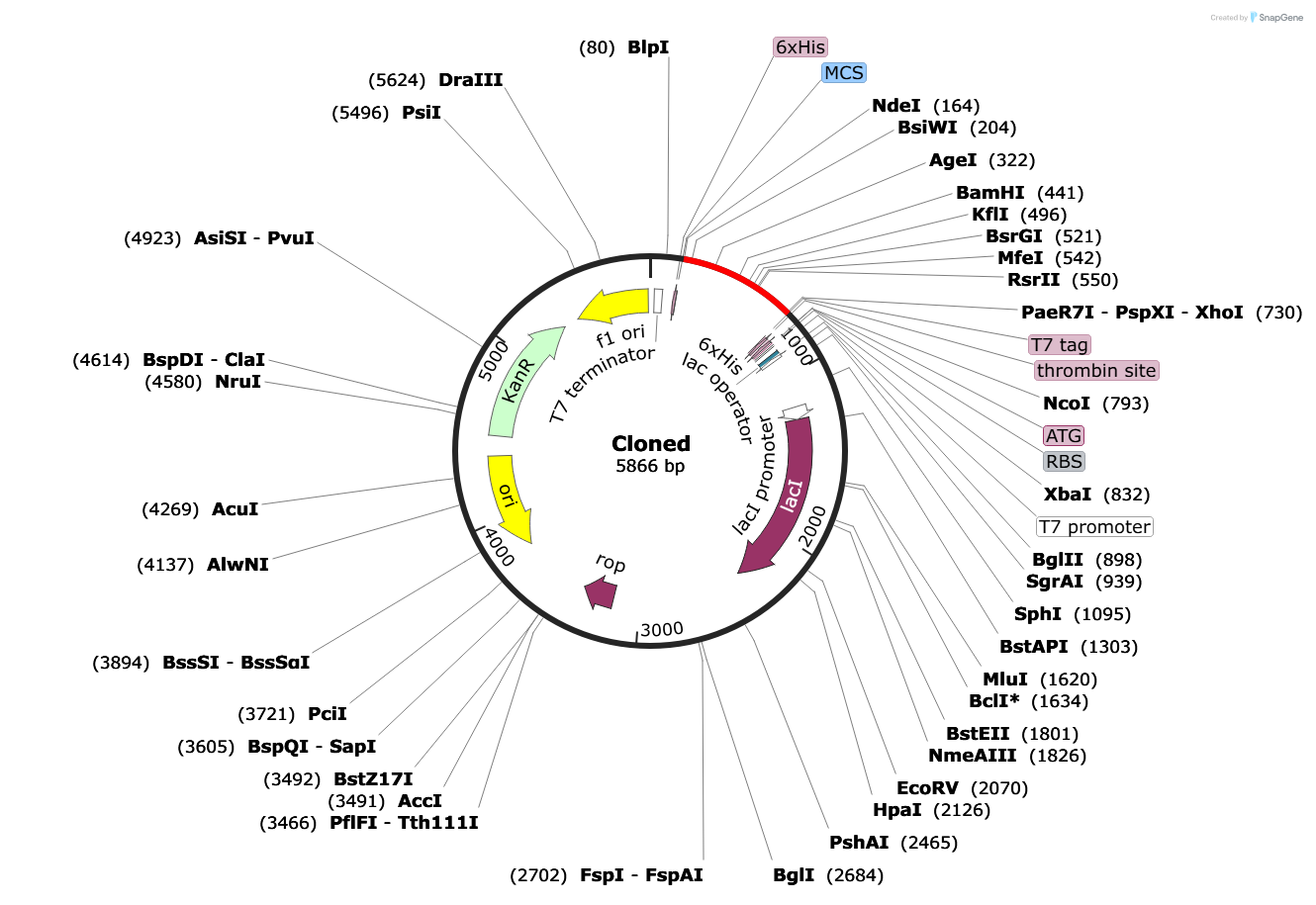


# **Supplementary Figure S1. In silico cloning and codon optimization.**

Schematic representation of the codon-optimized vaccine gene cloned into the pET-28a(+) expression vector between NdeI and XhoI restriction sites.

# **Figure 2. Transmembrane topology and surface accessibility analysis of selected antigenic proteins. Protein topology was predicted using the TMHMM v2.0 server to identify transmembrane helices and extracellular regions. The results demonstrate that the selected proteins possess significant surface-exposed domains, supporting their suitability as immunologically accessible vaccine targets.**

1.
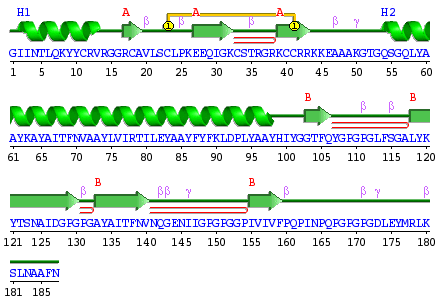


## **Figure 5. Structural modeling and validation of the multi-epitope vaccine construct.**

## **(A) Predicted three-dimensional structure generated using AlphaFold3. (B) Ramachandran plot analysis obtained using PROCHECK showing the distribution of residues in favored and allowed regions, indicating good stereochemical quality. (C) Secondary structure composition derived from PDBsum, illustrating the distribution of α-helices, β-strands, and random coils.**

C.

## **Figure 9. Recombinant expression and purification of the multi-epitope vaccine construct in *E. coli.* (A) SDS-PAGE analysis showing protein expression at the expected molecular weight (~21 kDa). (B) Western blot confirmation using anti-His tag antibody. (C) Size exclusion chromatography profile demonstrating monomeric protein distribution. (D) Dynamic light scattering (DLS) analysis indicating low polydispersity and high sample homogeneity.**
